# Parents of children with atopic diseases - experiences with care and the interaction with healthcare professionals over time

**DOI:** 10.1080/02813432.2024.2357794

**Published:** 2024-06-03

**Authors:** Gitte Færk, Elisabeth Søndergaard, Lone Skov, Jacob Pontoppidan Thyssen, Kirsten Skamstrup Hansen, Susanne Reventlow

**Affiliations:** a Department of Dermatology and Allergy, Copenhagen University Hospital – Herlev and Gentofte, Copenhagen, Denmark; b Centre for General Practice, The Research Unit for General Practice Slagelse/Copenhagen, and Section of General Practice, Department of Public Health, University of Copenhagen, Copenhagen, Denmark; c National Allergy Research Center, Copenhagen University Hospital – Herlev and Gentofte, Copenhagen, Denmark; d Department of Clinical Medicine, University of Copenhagen, Copenhagen, Denmark; e Department of Dermatology and Venereology, Copenhagen University Hospital – Bispebjerg and Frederiksberg, Copenhagen, Denmark; f Department of Pediatrics, Copenhagen University Hospital – Herlev and Gentofte, Copenhagen, Denmark

**Keywords:** Atopic dermatitis, comorbidity, continuity of patient care, food hypersensitivity, interview, qualitative study, parents

## Abstract

**Objective:**

To explore how the parents of children with atopic dermatitis and allergic diseases such as food allergy, allergic rhinoconjunctivitis, and asthma experience interactions with the Danish healthcare system over time.

**Design and methods:**

A qualitative design with individual in-depth interviews. The analysis was inspired by Systematic Text Condensation.

**Subjects:**

Eleven parents of children with atopic dermatitis and allergic diseases who received treatment at hospitals in the Capital Region of Denmark. The families had experiences of cross-sectoral patient care.

**Results:**

Despite having the same diseases, the children’s care pathways were very different. Mapping demonstrated the intricacy of care pathways for this group of children. We identified three aspects that impacted interaction with healthcare: responsibility, tasks, and roles. The families experienced care when the distribution of tasks and responsibilities associated with treatment and system navigation were consistent with both their expectations and their actual experiences. At the same time, families frequently experienced limited collaboration between healthcare professionals resulting in perceived fragmented care and an extended role for parents as care coordinators. Families felt cared for when healthcare professionals knew both their biomedical and biographical circumstances, and adjusted the level of support and care in accordance with the families’ particular needs, expectations, and evolving competences.

**Conclusion:**

We suggest that a possible pathway to improve care may be through a partnership approach as part of family-centered care, with general practitioners having a key role in helping to articulate the individual needs and expectations of each family.

## Introduction

It is incredibly frustrating to have a child with a disease or illness, for which there is no cure. It is just firefighting all the time.

Atopic dermatitis (AD) is a chronic pruritic, inflammatory skin condition that affects 10–20% of children [1]. Children with AD may have or develop other allergic comorbidities, such as allergic rhinoconjunctivitis (ARC), asthma, and food allergy [2]. The symptoms of AD and allergic diseases range from mild and transient to severe, with food allergy and asthma potentially being life-threatening. Children with uncomplicated allergies and atopic diseases are often treated in general practice [3]; however, specialties such as dermatology, pediatrics, otorhinolaryngology, and allergy can be involved, depending on disease manifestations, disease severity, and complexity, as well as regional variation in health care organization. An increasing number of individuals with allergy-associated diseases are seen in the hospital system in Denmark [3]. Evidence-based care is the cornerstone of clinical practice guidelines, which include systematic diagnostics, treatment, follow-up, and support for self-care [4]. There is, however, no disease management program for individuals with more than one atopic or allergic disease, involving several organs. As a result, there is no description of how responsibilities for communication and coordination are distributed when disease manifestations require treatment across multiple departments or healthcare sectors.

Patient-centered care, which is key in general practice and other medical specialties, is an approach where medical care is tailored to the individual patient and their family to enhance quality of life and health outcomes [5]. Previous research on experiences with healthcare have mainly focused on patients with a single disease [6–9]. A Swedish study on patient experiences with health professionals included participants with one or more allergic diseases, and pointed at inadequate information, talking past each other, and the level of care being determined by individual health professionals, which was considered a disadvantage [10]. Previous research, however, has not explored how the parents of children with AD and more complex disease patterns experience the care of their child and encounters with healthcare professionals.

This study explores how parents of children with AD and allergic diseases experience encounters with the healthcare system in the Capital Region of Denmark. The overall goal is to identify areas for improvement in care and to determine where new interventions could be developed.

## Material and methods

We chose a qualitative research design based on material from individual interviews with parents to allow for exploration of experiences regarding interactions with healthcare professionals when multiple atopic diseases are present.

### Informants

The informants are the parents of children with AD and at least one allergic comorbidity: food allergy, ARC, and asthma. They were selected based on responses to a questionnaire distributed between August 2020 and June 2021 at a pediatric and a dermatology department in a larger hospital in the Capital Region of Denmark [11]. This was part of a questionnaire study conducted among various hospital departments and practicing specialists treating children with atopic diseases, with a total of 301 participants included, of whom 279 completed the questionnaire. We recruited families with a child with AD and allergic comorbidities, who had experience with cross-sectoral care. Most of the children had food allergies. In total eleven families participated in the study (Table 1).

**Table 1.** Characteristics of children with atopic diseases and their families.

Family	Participating in the interview^a^	Age	Severity of AD over a 6 months period, parent reported, VAS 0–100 (0 is no symptoms and 100 is severe symptoms)^b^	Severity of food allergy over a 6 months period, parent reported, VAS 0–100 (0 is no symptoms and 100 is severe symptoms)^b^	Comorbidities^b^	Family history of atopic diseases^b^
1	Mother and father	5	63	16	Food allergy, ARC	Yes (AD, ARC, food allergy)
2	Mother	2	81	18	Food allergy	Yes (asthma, ARC, food allergy)
3	Mother	2	39	71	Food allergy	Yes (AD, ARC, food allergy)
4	Father	2	73	76	Food allergy, ARC	Yes (AD, ARC)
5	Father and partly mother	4	25	60	Food allergy	Yes (AD, asthma, ARC, food allergy)
6	Father	1	57	48	Food allergy	Yes (ARC)
7	Mother	5	41	–	Asthma, ARC	Yes (AD, asthma, ARC, food allergy)
8	Mother	1	72	-^c^	Allergic food reaction	No
9	Mother	3	7	83	Food allergy	Yes (ARC)
10	Mother	1	30	67	Food allergy	Yes (AD)
11	Mother and father	5	16	0	Food allergy	No

AD: atopic dermatitis; ARC: allergic rhinoconjunctivitis.

^a^We encouraged that the parent participating in the interview to be the one, who usually attended the consultations with healthcare professionals.

^b^Information from the questionnaire study (11).

^c^Was under investigation for food allergy at the time of the questionnaire completion.

For nine out of eleven children, all diagnoses were based on physician diagnoses documented in the child’s medical records from hospitals (secondary care). For two children, one of the diagnoses (one AD and one food allergy) was parent-reported as a physician-confirmed diagnosis. AD had to have been treated by a physician currently or within the previous two years. The severity of AD reported by parents varied among the children, with a mean severity of 46 on a visual analog scale (VAS) (Table 1) [11]. Similarly, the mean severity of parent reported food allergy was 49 (Table 1) [11]. The children’s ages ranged from one to five years. The study took place during the COVID-19 period, presenting a challenge in recruiting families for interviews, which led to a relatively homogeneous group. However, these families brought forth diverse experiences. In several cases, one parent originated from another country, contributing to a spectrum of experiences and expectations. The parents displayed a reasonably high health literacy.

### The interviews

Over a period of four months (June to September 2021), GF conducted one in-depth, semi-structured, individual interview with each of the participating families. The interviews followed an interview guide, which focused on the parent’s experience with the child’s diseases, their experience with care, the healthcare system, and the continuity of care during the period from diagnoses until the day of the interview (Supplementary Table S1). All interviews took place in the families’ homes. In eight out of the eleven interviews, only one parent participated (six mothers and two fathers); and in three, both parents participated (Table 1). The duration of the interviews was between 70 and 165 min. After each interview, GF wrote field notes regarding tacit information, impressions of the surroundings such as housing, home interior, presence of other individuals, and the cleanliness level (in accordance with parental statements), as well as immediate thoughts.

### Ethics

All participating families received both verbal and written information before the interviews, including information about the possibility of withdrawing their participation at any time. Both parents in each family gave written consent.

Danish Ethics Committees do not evaluate qualitative research. All patient-sensitive data are stored according to the regulations of the Danish Data Protection Agency and the study is registered (P-2020-659).

### Analysis

The interviews were audio recorded and transcribed verbatim by a research assistant. To support the analysis Nvivo (version 1.5.1) was used. Data were analyzed inspired by Kirsti Malterud’s Systematic Text Condensation [12], which is a thematic, cross-case, and explorative analysis. The technique has a pragmatic approach with four steps.

First, GF read all the text material, while ES and SR read selected interviews, after which the author group discussed preliminary themes. Second, based on the discussion, GF proceeded by systematizing the material and applying codes. During this step, all text was read line-by-line to identify text fragments relating to the selected codes. To outline the context for parents’ experiences with the care of their child and encounters with the healthcare system, we mapped the care pathways from each participating family. Third, we continuously produced text bites to test different strategies for working with the data, and two main themes were found to be particularly significant: 1) Care pathways – navigating the healthcare system; and 2) Responsibilities, decisions and roles. Each theme had subthemes to nuance the perspectives. Finally, at the end of the analytical process, the findings that included the identified themes were compared with the complete data to strengthen validity [12].

## Results

A pervasive aspect in the parents’ perception of their encounters with the healthcare system was their understanding of their child’s illness. Many parents perceived their child’s AD and comorbidities as something that could be treated and cured, and only over time an understanding was created that the situation was long-term and required continued and possibly lifelong handling.

The results will be presented in the two main sections ‘Care pathways – navigating the healthcare system’ and ‘Responsibilities, decisions and roles’.

### Care pathways - navigating the healthcare system

**Figure 1.**
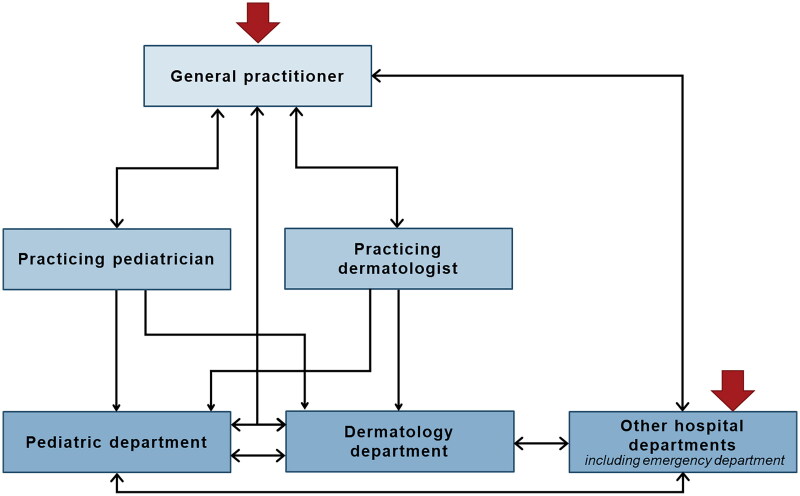
Illustrates existing routes through the Danish healthcare system (not exhaustive) based on the participating children with atopic diseases, and it visualizes the complexity in the continuity of care for this group of patients. Red arrows mark possible entries to the healthcare system.

**Figure 2.**
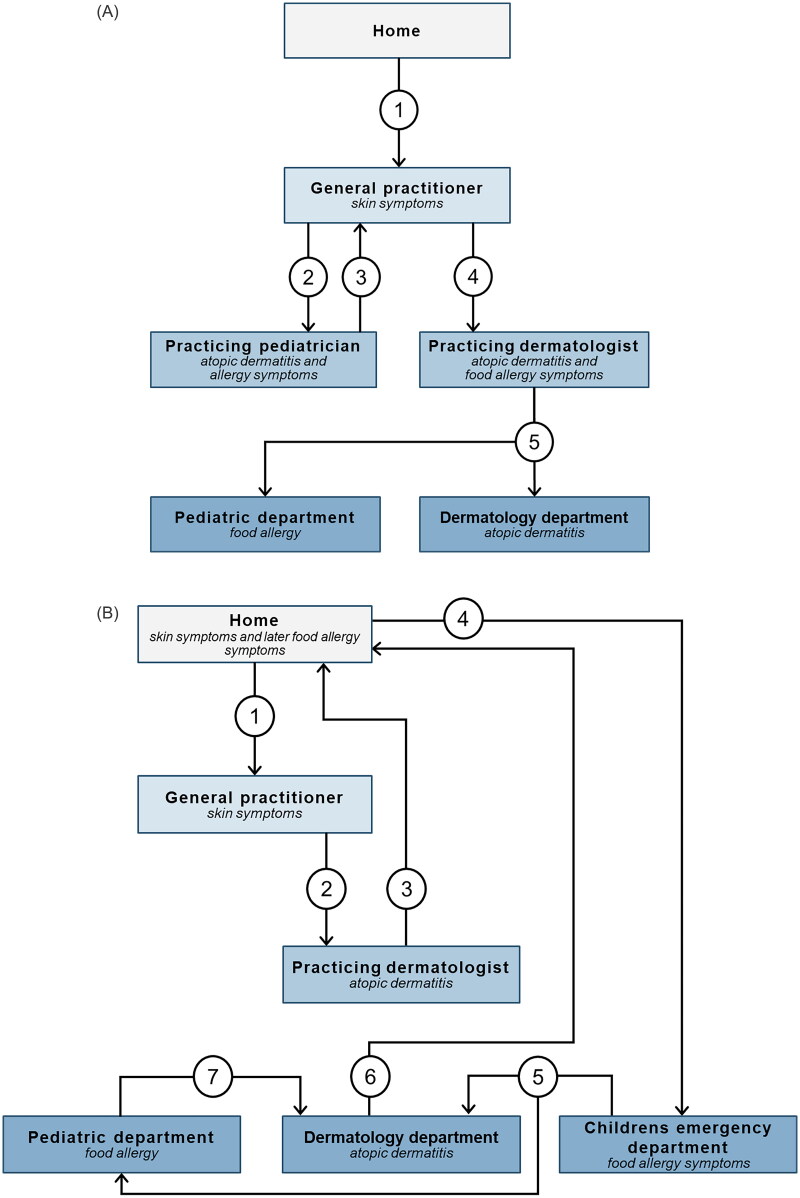
Two examples of care pathways. Each step of the care pathway is represented by a number.

Even though the children were diagnosed with the same diseases (AD and at least one allergic manifestation), the children’s paths through the healthcare system varied considerably. While care often began with the general practitioner (GP), a few families started their journey in hospital, due to an acute allergic reaction to a food item (Figure 1). Regardless of entrance point, care often involved several healthcare professionals from various specialties and/or locations in the healthcare system e.g. practicing specialists and hospital departments. The interaction with healthcare professionals was usually both sequential for a single disease and parallel for multiple diseases (Figure 2).

Our own doctor [GP] said that this was atopic dermatitis, so he wanted to send us to a dermatologist [practicing specialist]. So, we’ve been seeing a dermatologist, where we tried (sighed) more or less everything. Corticosteroid creams and uh, emollients… I feel like we tried so much… At the end the doctor was in doubt if it was something in the skin or an allergic reaction that caused this eczema to flare up. So, we were referred to a dermatology department at the hospital, and from there we were directed to the allergy clinic [part of the pediatric department]. So ehm, we have had kind of parallel visits these places.

The involvement of several different healthcare professionals often had a substantial impact on the parents’ perception of continuity of care. The parents appreciated the high degree of medical expertise at the hospital departments and valued the physician’s expert knowledge regarding the child’s diseases. They experienced challenges, however, with transitions of care and very limited collaboration between the different medical specialties. The parents expressed how they felt that they were passed on to the next professional, without any follow-up.

#### Limited communication/collaboration between specialties

Many parents experienced that the communication between the different medical specialties was based on short notes from the medical record and not directly *via* e.g. mail correspondence, telephone, or face-to-face. The communication between healthcare sectors was even more restricted and primarily conducted through the parents themselves. As a consequence, families had the impression that the physicians were often not briefed sufficiently on medical information from the other specialties, which meant that the parents had to explain the same things again and again.

Several parents described uncertainty about the health professionals’ areas of expertise and responsibility. A parent related that he had only recently been made aware that the pediatric department treated allergies (and asthma), but not AD/skin symptoms. This information about how diseases were segmented was puzzling to the family because, according to their understanding, there was a strong association between the diseases (food allergy can also cause skin symptoms). The pediatric department advised that the child needed a referral to a dermatologist and so they contacted their GP for the referral, but it was more complicated than anticipated:

Yesterday, I wrote an email to my GP, who then writes back to me and informs me that the hospital department made a note stating that I am advised to call my GP to get a referral [to a dermatologist], and my GP then says that I have to call the hospital department and get them to make a new note regarding the reason why they think we need a referral before he [can make the referral] – you know, it’s a bit [complicated]…

This quote demonstrates how the lack of direct communication between the hospital department and the GP resulted in a shift in the role the parents expected to fulfil, by adding new assignments for them to handle. Often parents became messengers and coordinators between two healthcare sectors and the transition of care became challenging. In addition, the example shows how difficult it is for families to understand both how healthcare is constructed, and how to navigate the system without prior knowledge or experience of it. This enhanced the perception of fragmented care among parents and, ultimately, it diminished the experience of continuity and coherence of care.

### Responsibilities, decisions and roles

#### Unclear roles and tasks – responsibility a heavy burden

Some parents felt their GP lacked knowledge and experience in dealing with AD and allergic diseases, especially regarding diagnosis, treatment guidelines, and referrals for specialized care. According to the parents, this led to a detour or delay before the families ended up at the right place in the healthcare system. At this early point, parents were still inexperienced with their child’s diseases, and they felt they had to take a larger responsibility for the care of their child within the healthcare system than they were comfortable with.

Overall, parents expected their GP to be the initiator, manager, and navigator, but often their experience was that these roles were transferred from the professionals to the parents. Some parents said they had to take the initiative to get a referral to a practicing specialist or hospital department, and they were then faced with the challenge of deciding which specific practicing specialist to consult. This task was not easy, because they had to figure out which specialist had expertise in atopic and allergic diseases in children and then wait for an appointment. One parent of a child with both AD and food allergy symptoms stated:

I actually think it was a bit confusing. I remember, I was at my GP’s, and he was like – yeah but eh, we can give you a referral, but somehow there was also a sense of, that I had to figure it out on my own – should I go to a pediatrician or should I go to a dermatologist? Where should I go – who was the best? I was googling pediatricians and dermatologists to figure out who was the best – was anybody specialized in this?

The parent in the example above had a good relationship with her GP and felt that the GP had listened to her needs. Nevertheless, she still had to be proactive and navigate between different specialists. Not being able to share this responsibility with a healthcare professional made the parent feel left alone. The parent did not anticipate this distribution of responsibilities and described the task as difficult because it was not aligned with her competences and knowledge at that point in time. In this situation, the parent experienced a discrepancy between her expected role and her assigned role and, consequently, it affected the experience of care.

Limited collaboration between medical specialties also affected the distribution of responsibility. Parents experienced that they had to put the different pieces of information together themselves and decide which treatment advice to follow. One parent described a situation where the treatment recommendations regarding antihistamine use from two different specialties were not aligned. The mother was placed in a dilemma where she had to make a decision based upon conflicting recommendations. On the one hand, following treatment recommendations resulted in restrictions, in that the child had to be closely monitored by the parents for 24 h after taking antihistamines. On the other hand, the child risked having an urticarial reaction. The responsibility became a (heavy) burden for the parent because she was not able to share it with healthcare professionals as expected. This distribution of responsibility and decision-making made the mother anxious and insecure.

Unclear tasks and ambiguity in the distribution of tasks between professionals and parents could make parents feel uncertain and confused. In the following example, the parents were supposed to bring food allergens to a skin prick test, but they did not realize it was their responsibility. Furthermore, the parents described how the task was above their competences.

Then you have a skin prick test with substances they have. They are lined up so you may just arrive [to the appointment] and they can test you with a variety of substances. But then at one appointment she [the healthcare professional] says oh you should have brought this and this yourself and I was like okay, but I know nothing about this … Then we had to be the experts on what substances are good to use for the test. Eh, and I was at a loss.

#### The healthcare professional taking responsibility for extended care: trust and creating a relationship

Parents described how they had experienced very different traditions and organizations within the various locations of the healthcare system. They all said that the experience of trust and a good relationship was related to how they felt the professionals cared for their child. Seeing the same physician each time supported this feeling. The material aspects of care varied only a little between the different physicians seen by the families, but the presence of a caring attitude varied a great deal, which influenced the families’ experience of care. Furthermore, meeting different physicians each time made the relationships more distant, as not every physician knew much about the child’s and the family’s biographies outside of the medical records. This limited the ability to talk about other aspects of the diseases such as consequences and frustrations. Some parents worried that their child was reduced to ‘a social security number (CPR-number) with a medical record’.

By comparison, one parent emphasized the relationship they had with their GP, where they always met a familiar face. The GP knew the family from previous visits, such as the preventive child health assessments and sibling visits. However, a good relationship and the GP’s knowledge of the family’s biographies could not stand alone, so if the material aspect of care was challenged due to the GP’s lack of knowledge about the diseases, then the families’ experiences could be impacted, and the interaction perceived as less caring.

The following example illuminates the significance of creating a relationship where all aspects of care are present. A child from one of the families had severe AD and the family was normally seen by the same physician each time they were at the department of dermatology. The physician became the key figure in the family’s interaction with the healthcare system. Overall, the parent described a caring and trustful relationship that also gave the family opportunities to discuss the challenges of the disease beyond the biomedical aspects.

At the department of dermatology we have been really happy with knowing [the name of the physician] and that we were able to share some of the challenges and frustrations concerning the disease. In addition to discussing the medical issues and looking at the eczema, we were able to discuss a number of other things… including the more difficult aspects, where we have been able to… to vent our frustrations to her.

Families interviewed in this study frequently encountered people in their everyday lives who were unaware of the impact of moderate to severe AD, including treatment burden, physical, and emotional consequences [13]. Thus, the physician’s accommodating attitude, feeling listened to and understood, and the possibility of talking about other aspects of life enhanced the experience of care.

#### The parents’ learning process and development of competences - equipped to take responsibility

Continuity of care is a continuous learning process for each family with different points of departure based on their prior knowledge. When the child first had symptoms, the parents were often inexperienced, both regarding the diseases and in navigating the healthcare system. The parents described a learning curve where they ‘learned the language’, while at the same time managed the diseases and their consequences, such as time-consuming AD treatment strategies and dietary restrictions. There was a consistent challenge between the parents’ perceptions and expectations and the achieved effects of care, i.e. how much treatment could improve the child’s situation and whether there were other possible treatments that would work better.

As part of acquiring skills and knowledge, the families received education from healthcare professionals, for example, in managing treatment strategies for eczema, including adjustments according to disease variations over time, observation/management of acute symptoms (food allergy), and general advice regarding eczema and allergy. The duration of hospital treatment was customized to the family’s needs and competences, and would last until they felt capable of managing the child’s disease:

In the beginning I talked with them [the hospital department] on the phone maybe once a week, but it was because she got an urticarial reaction from the food she ate, or she got diarrhea immediately after we gave her something [to eat]. I thought I had to call each time. I had to learn how to act, and it was difficult or at least a learning curve.

The individual families’ needs changed over time as they gradually became more competent and well equipped to handle their responsibility for care and disease management, mainly due to the education and support from healthcare professionals. However, even though a significant part of patient care for these diseases was focused on supporting and educating the parents (and the child) in disease management, one parent described how frightening it was to lose the safety net the department provided for them and their child, as well as the possibility of having to start over if they needed to contact the department again.

I believe that the connection [to a hospital department] has been the most important - somebody we could call. I might be, not afraid, but a bit worried, when we have completed the treatment at [name of the hospital department]. Because then we would have to start all over.

## Discussion

Care pathways for children with atopic diseases illustrated great complexity and they differed a lot even though the children had the same diseases. Families experienced care when there was coherence between the roles they expected to fulfil and the roles they experienced, including distribution of responsibility concerning treatment and navigation of the healthcare system. Yet families often experienced that their roles and responsibilities were extended. Families felt cared for when healthcare professionals had both biomedical and biographical knowledge about them and adjusted the level of support and care according to their individual needs and expectations.

### Strengths and limitations

A strength is the richness of material in the interviews; participating families shared detailed experiences both regarding the child’s diseases and care pathways.

The interviewer was a medical doctor, which helped in the interview setting because she had a thorough understanding of the diseases making it easier to ask in-depth questions. This is, however, also a potential limitation. The interview guide was developed in collaboration with the co-authors, who had backgrounds in anthropology, strengthening reflexivity, in terms of the researcher’s position and in data analysis.

All the children had AD. The findings might have differed for children with different combinations of atopic diseases that did not include AD. A limitation of the study is the lack of variance regarding the informants’ education level, family structure, and geographic location. At least one of the parents in each family had medium cycle (bachelor) or long cycle (master) higher education, and both parents lived together with their child. Still, the families represent the target group for the study [14], and they exhibited some variation within the experiences we explored. We argue that when there are challenges for well-educated and resourceful families, it is likely that the same or more challenges will be present for less resourceful families.

All participants resided in the Capital Region of Denmark. Corresponding care pathways in other regions could presumably differ due to variance in regional organization, including the number of practicing specialists and whether or not there is an allergy department at a regional hospital [3]. This may indicate that the findings are only partially transferable to different organizational settings. A Swedish study with caregivers from various regions with differences in the organization of healthcare found similar challenges in the care of children with allergic diseases [10], indicating that these challenges are widely applicable. Despite residing in the same region, the children’s care pathways in this study still differed a lot.

### Comparison with existing literature

Previous studies have mainly addressed experiences with care and healthcare professionals when a single disease is present [6–8, 15]. According to a recent review, parents of children with AD experienced ‘involuntary autonomy’, which was characterized as being responsible for self-management and feeling left by themselves, as a result of conflicting advice and inadequate information and support from healthcare professionals [6].

Our study shows how a group of children with multiple associated diseases challenges parents’ experiences with continuity of care, which adds an extra layer of complexity to their interaction with healthcare professionals. For some families, this means a very complicated process and uncoordinated contact with several healthcare professionals, which is comparable to the experience of many adult patients with multimorbidity [16]. A possible explanation for fragmented care can be that the secondary and tertiary (specialist) levels of the healthcare system are mainly organized around single diseases or organs, which provides a high degree of medical expertise, but may result in fragmented care when patients have more than one chronic disease [16]. This is likely applicable to healthcare systems in other countries as well.

Differences in GPs’ knowledge of referral pathways and treatment options for AD and allergy may also be part of the reason. A British study by Le Roux et al. examined GPs’ perspectives and their experiences with AD [17]. The GPs described gaps in their knowledge about optimal treatment due to limited training, and that AD had a low priority compared to other long-term diseases [17]. In accordance with these findings, the parents in our study reported that their GP frequently lacked knowledge about available treatments and possible referral pathways when the diseases required specialized care, and this also resulted in the parents’ extended roles as managers and navigators.

On the other hand, the organization of the healthcare system was puzzling to families who viewed the diseases as strongly connected and therefore, often experienced that their views were incompatible with those of healthcare professionals. Divergent views concerning understandings of AD between healthcare professionals and parents are also described in a study by Powell et al. where GPs’ main focus was the management of the disease, while parents, in their ‘search for a cure’, saw allergy as a probable cause of the child’s AD [7].

At first glance, the families in this study looked quite similar based on the selection criteria, but our results showed that they had very different needs and expectations, due in part to their prior knowledge and experiences with the diseases and the healthcare system. This emphasizes the importance of patient-centered care with the possibility of individual adaptations, which is in accordance with findings from other studies [6]. However, the need for individual adaptations and the complexity of multiple long-term diseases were part of the explanation as to why a previous study among adults with multiple diseases found limited clinical applicability of disease management programs that only include a single disease [4]. The concept of care used by Annemarie Mol entails individual adaptations with the use of knowledge and technology to support patient treatment [18]. She uses the concept of ‘tinkering’ to explain the dynamic process which involves both the healthcare professional and the patient/parents where, in collaboration, they compose and recompose the optimal course of treatment fitted to the individual patient and family [18]. In relation to the learning process among the participating families, they described situations where the course of treatment had been tailored to meet the individual family’s needs including additional support, educational initiatives, and individually adapted duration of treatment courses. Yet, as previously mentioned, there were other situations where, according to the parents, tinkering could be improved. Tinkering has usually concerned interaction with just one healthcare professional and the examples of it mentioned by the parents in this study were also all related to the care pathway for a single disease at one location (e.g. hospital department). Implementation of tinkering in the entire care pathway across different sectors and specialties requires a coordinating healthcare professional and interorganizational integration of care [19]. This is a role the GP has when it comes to chronic diseases in adults [20].

According to Fawcett et al. healthcare professionals need to be aware of each family’s needs, beliefs, and expectations as part of the partnership approach to promote patient satisfaction and improved health outcomes [9]. In another study by Smith et al. about how to achieve actual collaboration [21], they emphasize how healthcare professionals must assume responsibility for establishing partnerships with parents, with ongoing flexible adjustments to meet the changing support needs as the parents’ role evolves from passive to active [21]. They suggest that by assisting parents to interpret and navigate the healthcare system, collaborative practices could lead to better access and care coordination, which is particularly important when a child has more than one disease [21].

We found three major aspects that influenced parents’ interaction with healthcare professionals: responsibilities, tasks, and roles. We argue that care is experienced when there is coherence between the expected and the experienced roles and the distribution of responsibility and tasks. In these situations, parents’ needs are recognized by health professionals and addressed accordingly [22]. Improving the interaction between families and healthcare professionals, and hence improving the experience of care, requires continuous work including a caring attitude and ongoing clarification of mutual expectations and the distribution of assignments and responsibilities [22]. In a systematic review, Ring et al. emphasize that plans should be tailored individually according to patient and caregivers’ needs, as well as paying attention to the bigger picture associated with having a child who lives with long-term diseases [23].

In healthcare, it is not necessarily possible to change the distribution of roles and responsibilities, but a way to increase the experience of care could be to articulate roles, responsibilities and tasks, including the distribution and alignment of expectations, during the first consultations and then to readdress these in later consultations, to accommodate changes in the parents’ needs as their competences develop over time. Fawcett et al. also argue that a way to ensure successful collaboration between healthcare professionals and parents is by sharing responsibility [9].

## Conclusion and implications for practice

The children’s care pathways and the parents’ needs and expectations differed a lot, even though the children had the same atopic diseases. Many parents experienced limited collaboration between healthcare professionals resulting in perceived fragmented care and an extended parental role as coordinators of treatment between healthcare professionals and settings. Care was experienced when there was coherence between the expected and the experienced roles and distribution of responsibilities and tasks.

We suggest that a possible route to improved collaboration and interaction between healthcare professionals and parents of children with AD and allergy could be through a partnership approach as part of family-centered care. Furthermore, all healthcare professionals involved in care of children with complex diseases should possess and impart relevant knowledge to better align with each child and the family and meet their specific informational needs. This involves health professionals being aware of and helping to articulate individual needs and expectations. It requires continuous adjustment according to changes in parents’ needs as their knowledge and competences increases. To improve coherence in care, our study also underlined the necessity of direct information sharing between medical specialties and healthcare sectors.

General practice has a central role to play when children have chronic diseases due to its facilitator and coordinator function. Further research is required which could look at the development of a new intervention that utilizes the GP’s active role in the management of chronic disease in children, the transitions of care, and how to build partnerships with parents and collaborators across the healthcare system.

## Supplementary Material

Supplemental Material
