# Supplementary material for: Parents of children with atopic diseases - experiences with care and the interaction with healthcare professionals over time
Source: Scand J Prim Health Care. 2024 Jun 3;42(4):550–9. doi: 10.1080/02813432.2024.2357794 (PMC11552268; doi:10.1080/02813432.2024.2357794)
Supplement: Supplemental Material [file IPRI_A_2357794_SM8519.docx]

**Supplementary table 1**

Interview guide for parents of children with atopic diseases

| **Theme** | **Questions** |
| --- | --- |
| **Experiences with atopic dermatitis and associated diseases** | |
| Diseases and duration | - How old was the child when he/she first experienced atopic dermatitis/eczema? - What other diseases does the child have in addition to atopic dermatitis/eczema? - How old was the child when he/she developed food allergy/hay fever/asthma? |
| Daily life – the influence of the disease on everyday life  *Both the child’s and the family’s perspective* | - What was the initial experience leading up to the first contact with a medical doctor (e.g., your GP)? Symptoms, duration, discomfort, concern. - How is the child currently coping – to what extent is the child's health affected (severity)? - Do the child’s diseases (atopic dermatitis, food allergy, hay fever, and/or asthma) influence the child’s daily life? If yes, could you describe how his /her diseases (atopic dermatitis, food allergy, hay fever, and/or asthma) affect the child’s daily life? - Is the child affected or unaffected? Are there any limitations? Are there activities that the child cannot participate in (possibly without parental supervision, e.g., playdates, sports)? Are there any precautions you need to take? - Do the child’s diseases (atopic dermatitis, food allergy, hay fever, and/or asthma) influence the family’s daily life? - If yes, could you describe how the child’s diseases (atopic dermatitis, food allergy, hay fever, and/or asthma) affect the family’s daily life? |
| Regarding daycare institution/school and family/friends | In relation to daycare/preschool/school   - Do you feel that daycare/preschool/school is equipped to handle your child's condition/allergy? (Are you aware if they have received training, for example, in administering an epinephrine auto injector, acute asthma medication, etc., e.g., by a nurse from the pediatric asthma and allergy clinic) - Do you experience that consideration is given (to atopic dermatitis and allergy) in the daycare/preschool/school? - Are you comfortable leaving your child there? Do you feel confident that they can handle it if the child experiences acute symptoms (e.g., allergic reaction after eating, asthma attack)? - What is your experience when you are with family/friends, for example, at a birthday party? Do you feel safe, and are considerations made? - Babysitting (both by family and someone outside the family) |
| Own initiatives | - What steps have you taken in relation to your child's conditions such as atopic dermatitis and food allergy, hay fever, or asthma? |
| **Care pathway** | |
| Experiences with care pathways, preferably with concrete examples | I/we are interested in hearing about the care pathway – the places you have visited to identify your child's problems/diseases – and the treatments that have been given.   - Could you describe it for me? - Which healthcare providers have you visited for atopic dermatitis? For example, your GP, practicing specialist, private hospital, pediatric hospital clinic, pediatric asthma and allergy hospital clinic, dermatology hospital clinic. - Which healthcare providers have you visited for food allergy/hay fever/asthma? - Where is the child currently being treated for the diseases? |
| Parental roles | - Who typically attends medical appointments? - Have you established agreements throughout the process – concerning when to respond to symptoms, concerns/worries, expectations and recommendations from medical doctors/nurses (healthcare guidelines)?   Medication:   - Who is responsible for treatment of the child, such as topical steroids, asthma medication, or hay fever medication? - Have you ever received a prescription for the child that you did not pick up? Do you follow the treatment prescribed by the medical doctor, including the instructions for use (e.g., applying cream, taking inhalation medication, etc.)? Or have you made any changes to the treatment? - Do you have any concerns about the treatment, such as side effects? |
| Previous encounters with the healthcare system and sector transitions | The child has previously consulted a GP or practicing specialist for atopic dermatitis and other allergic diseases   - How was the transition from your GP to a practicing specialist or hospital experienced?   The child has previously been seen in various hospital departments, such as the pediatric clinic and pediatric asthma and allergy clinic:   - How was the transition between different departments or clinics at the hospital experienced?   Expectations   - What were your expectations regarding the care and treatment provided by your GP, practicing specialist, and hospital? - Was the care pathway as you expected? |
| Communication | - How has the communication from the healthcare system (GP/practicing specialist/hospital department) to you been? - Do you feel that the medical doctors and nurses you have encountered along the way have been prepared to understand you (needs, concerns, knowledge) and your child's diseases? - Do you feel that the medical doctors and nurses have listened to you (concerns, fears, challenges, frustrations, good experiences, needs, wishes, expectations, and questions)? - Are you of the impression that there has been a clear plan regarding investigation and treatment (both current location and previous locations)? At the GP, practicing specialist, and hospital. - Has it also been clear what should happen next when transitioning from one medical doctor to the next? - Have you had sufficient time during your visits to your GP, practicing specialist, or hospital physician? Have all your questions been answered? - Do you feel that the medical doctor has shown understanding of your situation regarding treatment (the burden of treatment from multiple medical doctors - the effort and energy you have to put into it)? - Has the medical doctor involved you in creating the treatment plan/goals, or has the medical doctor simply instructed you on what to do? (involvement) |
| Intersectoral collaboration and communication during simultaneous courses of treatment | - How do you find the experience of visiting one medical doctor/location for atopic dermatitis and another medical doctor/location for the other allergic diseases? (Go to different locations simultaneously or sequentially) - What impression do you have of the communication and collaboration between two hospital departments or hospital department and practicing specialist/GP? |
| Future | - How do you envision the care pathway moving forward? What expectations do you have? - Is there anything that worries you? - What is most important to you? |
| Optimization of the care pathway | - What is most important to you in relation to your child's diseases and care pathway? - Have there been any challenges in your child's care pathway? Preferably with examples - Can you think of anything that could have made your child's care pathway better? |
|  | - Is there anything concerning your child's diseases that you have been thinking about but we have not addressed yet? |

GP – general practitioner
